# Serum Uric Acid level in the severity of Congestive Heart Failure (CHF)

**DOI:** 10.12669/pjms.332.11779

**Published:** 2017

**Authors:** Adnan khan, Mohammad Hassan Shah, Sarbiland khan, Umama Shamim, Sanan Arshad

**Affiliations:** 1 Adnan Khan, House Officer, Rehman Medical Institute, Peshawar Pakistan; 2 Mohammad Hassan Shah, Final Year Students (MBBS), Rehman Medical College, Peshawar Pakistan; 3 Sarbiland Khan, Final Year Students (MBBS), Rehman Medical College, Peshawar Pakistan; 4 Umama Shamim, Final Year Students (MBBS), Rehman Medical College, Peshawar Pakistan; 5 Sanan Arshad, Final Year Students (MBBS), Rehman Medical College, Peshawar Pakistan

**Keywords:** Heart failure, Risk factor, Uric acid

## Abstract

**Background and Objective:**

It has been observed that in a clinical condition like hypoxemia there is an increase in the serum Uric acid level. The objective of our study was to find out the relationship between serum uric acid levels in the severity of Heart failure.

**Methods:**

We analyze 285 patients with a diagnosis of Congestive heart failure admitted in Lady Reading Hospital Peshawar from March 1^st^ to August 2016. Age group of patients was 17- 67 years. New York Health Association (NYHA) scoring were used to access the severity of Congestive Heart Failure. Serum UA level >7.0 mg/dl was considered high.

**Results:**

Total 285 patients with CHF were analyzed with a mean age of 54±2.8 years in which males were 65.96% and 34.03% were female. 40% were in class II of New York Health Association (NYHA), 32.63% in class III and 25.61% in class IV and 1.75% were in class I. Out of 285, 59.29% met the definition of hyperuricemia. In which 83.43% were male and 16.57% were female. Most of the Hyperuricemic patients 62.13% were in age group of 51- 60 years, with a mean age of 57±4.5 years. We found a significant correlation between uric acid level and BNP (p= <0.001), and use of diuretics (p=<0.001). 34.93% of the Hyperuricemic CHF patients were in NYHA III and NYHA IV whose SUA was above 8 mg/dl as compared to 31.57% Hyperuricemic CHF patients whose SUA was below 8 mg/dl.

**Conclusion:**

High serum Uric acid was observed in 59.29% of patients with CHF. The observed significant correlation between UA level and some established prognostic markers in these patients may indicate that serum UA could provide additional prognostic information in this population. SUA as a marker can be measured anywhere at a low cost to help identify high-risk patients with CHF. Lowing uric acid is expected to be a new approach for prevention and therapy of HF.

## INTRODUCTION

Uric acid (UA) is the end product of purine breakdown and is excreted by the kidneys. The enzymes responsible for the uric acid breakdown and production is xanthine oxidase (XO) and xanthine dehydrogenase. Both enzymes catalyze the oxidation of hypoxanthine to xanthine; this is a key enzyme in purine metabolism and the main contributor to the generation of oxygen free radicals which ultimately leads to increased oxidative stress. In addition, oxidative stress along with nitric oxide disproportion could intensify inflammatory pathways resulting in further increase in cytokine production.^1^ The normal serum uric acid levels range from 2.4–7.4 mg/dL in males whereas in females it ranges from 1.4–5.8 mg/dL.^2^

Chronic heart failure (CHF) is a leading etiology for both morbidity and mortality on a global level, resulting in an increase in both prevalence and health care costs. Recently, our understanding has changed from a mere hemodynamic condition to a much more complicated approach, including neuroendocrine and immune activation. Not only is the cardiovascular system damaged in the long run course of heart failure, but together with peripheral tissues and organs also result in the production of symptoms along with the progression of the disease.

Epidemiological studies have found an association between increased serum uric acid (UA) levels to an elevated vascular event rate and mortality in patients with hypertension, diabetes and prior cardiovascular disease.^3^,^4^

More than 550,000 new cases of heart failure are now diagnosed each year in America.^5^ Heart failure (HF), which is an increasing public health problem with skyrocketing healthcare costs and high mortality rates, affects around about 1–2% of the adult population in developed nations.^6^ Increased UA levels are also common in chronic heart failure (CHF). The relationship between serum uric acid and cardiovascular disease has gained a lot of attention over the years.^7^

Serum uric acid may be an important indicator for predicting people with preexisting heart failure.^8^ Not many studies have been conducted that evaluated increased serum uric acid (UA) levels as an independent risk factor for heart failure among the general population. The objective of our study was to find out the relationship between serum uric acid levels in the severity of Heart failure.

## METHODS

The study included 285 patients, consecutively admitted to Cardiology Department, Lady Reading Hospital Peshawar from 1^st^ March 2015 to August 2016. They were between 17 to 67 years of age. Those with Secondary hyperuricemia, like drugs, malignancies, uremia and other conditions with rapid cell turnovers live psoriasis were excluded from the study.

Heart Failure was diagnosed by trained cardiologists following current guidelines.^9^ The purpose and benefits of the study along with maintenance of professional secrecy were explained to the patients and a written informed consent was obtained.

**Fig.1:**
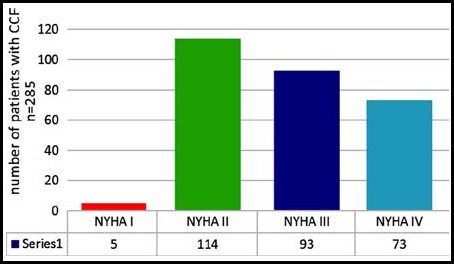
Shows severity distribution of congestive heart failure in our study according to NYHA classification

All patients were subjected to a detailed history and examination. The severity of Congestive Heart Failure was assessed using New York Health Association (NYHA) classification. Fig-1. Serum uric acid level was measured, after overnight fasting by enzymatic methods using the chemical analyzer. Hyperuricemia was defined as a serum UA level >7.0 mg/dl. From all patients, 5 cc of blood was taken under strict aseptic techniques and was sent to the hospital laboratory on the same day. Serum uric acid level was measured under the supervision of a pathologist. All the above-mentioned information including name, age, gender, and address were recorded in the study Performa.

### Data analysis

Data collected was entered in SPSS 22. Mean±SD was calculated for a continuous variable like age and serum uric acid levels and categorical variable like gender was expressed as frequencies and percentages. Chi-square test was applied with P value taken as < 0.05 as significant.

## RESULTS

A total of 285 patients with Congestive Heart Failure were evaluated with age group ranging from 17-67 years with a mean of 54±2.8 years in which males were 188(65.96%) and 97(34.03%) were female. Out of total 285, 169(59.29%) met the definition of hyperuricemia. Table-I shows baseline characteristics of patients with CCF.

**Table-I:** Showing baseline characteristics n=285.

*Variables*	*Hyperuricemic (n=169)*	*Normouricemics (n=116)*	*P value*
Male	141(83.43%)	47(40.51%)	
Female	28(16.56%)	69((69.48%)	
BMI	24.6±07	22.9± 09	0.168
Systolic Blood Pressure (mm Hg)	153±32	156±32	
Diastolic Blood Pressure (mm hg)	87±21	86±19	
Mean Heart Rate	73.9±3.6	67.5±2	0.239
BNP (pg/ml)	206±13	79.8±9	<0.001
***Risk factors***		
Hypertension	113(66.86%)	75(64.65%)	0.432
Diabetes Mellitus	78(46.15%)	41(35.34)	0.064
Hyperlipidemia	37(21.89%)	28(24.13%)	0.086
Current Smoking	13(7.69%)	7(6.03%)	0.932
***Drugs***		
Beta Blocker	39(23.07%)	21(18.10%)	0.187
Diuretics	79	43	<0.001
ACEI/ARBS	163	103	0.643
Digitalis	19	17	0.051
***Etiology***			
Ischemic heart disease	88	69	0.167
Valvular heart disease	76	74	0.976

From the total 285 patients with CCF, 17 were in age group less than <18 years, 35 were in age group of 18-40 years, 58 were in age group of 41-50 years while 146 were in age group of 51-60 years and 29 were in age group >60 years.

## DISCUSSION

We demonstrated that 59.29% of these patients had elevated serum uric acid levels. Increased serum uric acid levels have been constantly reported in patients with CHF, and recent clinical data supports the possibility that uric acid levels provide important prognostic information alone and in combination with other indicators of heart function and patients’ functional status in this group.^10^ Numerous studies have reported that hyperuricemia indicates a higher relative risk of all-cause mortality in patients with CHF, independent of other risk factors. In a recent study, high uric acid levels increased all-cause mortality in patients with both acute and chronic HF.^11^ which, in turn, increases XO activity and subsequently SUA levels. The purpose of this study was to perform a meta metaanalysis to evaluate the evidence supporting SUA as a predictor of all-cause mortality in patients with heart failure (HF) The Higher uric acid level was associated with long-term adverse outcomes in these patients.^12^ Several researchers have shown a correlation between increased serum uric acid levels in CHF and morbidity and mortality.^11^,^13^,^14^ which, in turn, increases XO activity and subsequently SUA levels. The purpose of this study was to perform a meta-analysis to evaluate the evidence supporting SUA as a predictor of all-cause mortality in patients with heart failure (HF)

It has been shown that in patients with mild to moderate CHF, elevated serum UA levels are strongly related to death, and this correlation is independent of Chronic Heart Failure severity and impaired renal function. In the same study, hyperuricemia was said to have predicted exercise intolerance and was an indicator of inflammatory activation in CHF. Serum UA levels increased extremely alongside with CHF severity expressed as NYHA class.^15^

**Table-II:** Serum uric acid levels and severity of congestive heart failure: n=285.

*SUA (mg/dl)*	*NYHA I*	*NYHA II*	*NYHA III*	*NYHA IV*	*Total*

	*n (%)*	*n (%)*	*n (%)*	*n (%)*	*n (%)*
<6	3(1.05%)	40(14.03%)	27(9.47%)	5(1.75%)	75(26.31%)
6 - 8	1(0.35%)	37(12.98%)	28(9.82%)	24(8.42%)	90(31.57%)
8.1 - 12	1(0.35%)	36((12.63%)	30(10.52%)	30(10.52%)	97(34.93%)
>12	0(0%)	1(0.35%)	8(2.80%)	14(4.91%)	23(8.07%)
Total	5(1.75%)	114(40%)	93(32.63%)	73(25.61%)	285(100%)

Mean serum uric acid (SUA) levels are 7.79 mg/dl with SD ± 2.47.

In our study, SUA was significantly higher among symptomatic Congestive Heart Failure patients than in asymptomatic patients with 34.93% of the Hyperuricemic CHF patients were in NYHA III and NYHA IV whose SUA was above 8 mg/dl as compared to 31.57% Hyperuricemic CHF patients whose SUA was below 8 mg/dl. This fact may help to identify asymptomatic patients in follow-up. We also found that mean SUA levels increased significantly with NYHA class. These findings suggest that SUA can be a potential biomarker for the assessment of response to therapy to either medication or surgical intervention in follow-up. In compliance with earlier studies in patients with heart failure, SUA was significantly correlated with LVEDD, LVESD, and LVEF. Therefore, we believe SUA levels may be useful to assess the extent of LV remodeling.

Serum uric acid has also been shown to be inversely related to the magnitude of functional capacity and maximal oxygen intake.^16^ Among patients with CHF, the SUA concentrations are associated with greater activity of superoxide dismutase and endothelium-dependent vasodilatation.^17^

While the findings of our study do not clarify the possible causal relations between assessment in these measures and disease intensity, they are consistent with our recent demonstration that SUA levels indicate mortality in patients with chronic heart failure.

Another potential pathophysiological link between elevated serum uric acid levels and heart failure might be via inflammation. Asymptomatic hyperuricemia is a pro-inflammatory condition affiliated with elevated levels of serum markers of inflammation, such as C-reactive protein, interleukin-6 and neutrophil count.^18^

The significance of our observation lies in its use for developing a risk prediction rule for heart failure. Although the reviews we have conducted does raise the possibility of primary prevention of heart failure, the literature is inconsistent on whether a decrease in serum uric acid will result in measurable clinical benefit to those with well-established heart failure.^19^

Some even argue that increased SUA occurring as a result of diuretic use may have a beneficial role in itself.^20^ In patients with heart failure, high levels of serum uric acid are highly indicative of mortality and are important in suggesting the need for heart transplantation.

### Limitations of the study

Limitations apply to our analysis. Our data on serum uric acid are essentially left truncated, that is we know the extent of hyperuricemia but not the period of hyperuricemia. Also, the distribution of serum uric acid concentrations among males and females was different, the previous having elevated concentrations.

## CONCLUSION

High serum uric acid was observed in 59.29% of patients with CHF. Serum uric acid levels can help to distinguish patients with asymptomatic congestive heart failure (CHF) patients from symptomatic patients. The observed significant correlation between UA level and some established prognostic markers in these patients may indicate that serum UA could provide additional prognostic information in this population. Such a simple marker that can be measured anywhere at a low cost to help identify high-risk patients with CHF. Lowing uric acid is expected to be a new approach for prevention and therapy of HF.
